# The interrelationship between phagocytosis, autophagy and formation of neutrophil extracellular traps following infection of human neutrophils by *Streptococcus pneumoniae*

**DOI:** 10.1177/1753425917704299

**Published:** 2017-04-11

**Authors:** Ihsan Ullah, Neil D Ritchie, Tom J Evans

**Affiliations:** Institute of Infection, Immunity and Inflammation, University of Glasgow, Glasgow Biomedical research Centre, Glasgow, UK

**Keywords:** Autophagy, neutrophil extracellular trap, pneumolysin, *Streptococcus pneumoniae*, bacterial killing

## Abstract

Neutrophils play an important role in the innate immune response to infection with *Streptococcus pneumoniae*, the pneumococcus. Pneumococci are phagocytosed by neutrophils and undergo killing after ingestion. Other cellular processes may also be induced, including autophagy and the formation of neutrophil extracellular traps (NETs), which may play a role in bacterial eradication. We set out to determine how these different processes interacted following pneumococcal infection of neutrophils, and the role of the major pneumococcal toxin pneumolysin in these various pathways. We found that pneumococci induced autophagy in neutrophils in a type III phosphatidylinositol-3 kinase dependent fashion that also required the autophagy gene *Atg5.* Pneumolysin did not affect this process. Phagocytosis was inhibited by pneumolysin but enhanced by autophagy, while killing was accelerated by pneumolysin but inhibited by autophagy. Pneumococci induced extensive NET formation in neutrophils that was not influenced by pneumolysin but was critically dependent on autophagy. While pneumolysin did not affect NET formation, it had a potent inhibitory effect on bacterial trapping within NETs. These findings show a complex interaction between phagocytosis, killing, autophagy and NET formation in neutrophils following pneumococcal infection that contribute to host defence against this pathogen.

## Introduction

*Streptococcus pneumoniae* (the pneumococcus) is a Gram-positive bacterium that is a major cause of community-acquired pneumonia and otitis media, as well as invasive bacteraemia and meningitis. Despite antibiotics and vaccine development, the infection remains a highly significant cause of morbidity and mortality worldwide.

One of the most important innate defence mechanisms against pneumococci are neutrophils, which rapidly migrate to sites of colonization and infection.^1–3^ Depletion of neutrophils in a mouse model permits invasive disease to develop, and neutrophil killing of pneumococci contributes to subsequent adaptive immune responses.^4^ A number of immunodeficiencies and haematological malignancies associated with neutropaenia are also risk factors for invasive pneumococcal disease.^5^ Taken together, these findings demonstrate the importance of neutrophils in host defence against pneumococcal infection.

Neutrophil killing of pneumococci is not dependent on reactive oxygen production but principally on serine proteases.^6^ Recently, a novel mechanism of neutrophil-mediated bacterial killing has been described, known as neutrophil extracellular traps (NETs).^7,8^ NETs are formed following neutrophil stimulation, and are formed of an extracellular web of DNA to which nuclear constituents such as histones bind, as well as neutrophil granule proteins. Although originally thought to result from neutrophil death, evidence now suggests that NETs can form without substantial neutrophil necrosis.^9,10^ Studies on pneumococcal interactions with NETs suggests that the microbe is captured but not killed by NETs *in vitro* and *in vivo.*^11^ A bacterial endonuclease allows the pneumococci to escape from the NETs; capsule and d-alanylation of lipoteichoic acids also protect pneumococci from NETs. Other studies have suggested that NET formation in pneumococcal pneumonia may be associated with enhanced lung damage.^12,13^

The mechanism of control of NET formation following interaction with pathogenic bacteria is not entirely clear. A variety of pathogens, as well as bacteria components such as LPS and cytokines such as IL-8, can stimulate NET formation by neutrophils,^8^ as well as purified pneumolysin, one of the most important pneumococcal toxins.^14^ A key intermediate required for NET generation is the production of reactive oxygen intermediates, which are lacking in neutrophils from patients with chronic granulomatous disease, thus preventing NET formation in this condition.^15^ Other studies have also implicated the autophagy pathway in NET formation.^16–18^ Autophagy is a highly conserved cellular mechanism that allows for degradation of cytoplasmic material and organelles under certain conditions, allowing for recycling of their constituents. However, it also plays an important role in infection and inflammation, playing a role in killing of phagocytosed microbes and down-regulating activation of the inflammasome.^19–21^

How these different pathways of intracellular killing, pneumolysin action, NET formation with bacterial entrapment and induction of autophagy combine in the interaction of neutrophils with pneumococci is not clear. We thus set out to determine the role of each of these processes in the response of human neutrophils to the pathogenic pneumococcal strain D39. We found that D39 induced autophagy in human neutrophils in a classic *Atg5* dependent fashion. Pneumolysin reduced the rate of phagocytosis of pneumococci by neutrophils, but enhanced the rate of intracellular killing. Autophagy enhanced phagocytosis but reduced the rate of intracellular killing. NET formation was dependent on autophagy, but not affected by pneumolysin. However, this toxin significantly reduced the numbers of bacteria adherent within the NETs.

## Materials and methods

### Materials

Immunofluorescence analysis of LC3 was performed using a polyclonal rabbit Ab (APG8B; Abgent, San Diego, CA, USA); Western blotting was performed using a polyclonal rabbit Ab (NB100-2220; Novus Biological, Abingdon, UK).

### Bacterial strains

D39 and D39ΔPly mutant were kindly supplied by Professor T. Mitchell, University of Birmingham.^22^ Bacteria were streaked on blood agar plates from frozen stock and grown overnight (16 h) in 5% CO_2_. Purified isolated colonies were transferred to sterile brain–heart infusion broth (Cat. CM1135; Thermoscientific Oxoid, Basingstoke, UK). Bacteria were incubated at 37℃ for about 6–8 h until grown to the mid-log phase (OD600, 0.4–0.6) immediately prior to use. Bacteria were collected by centrifugation at 3500 *g* at 4℃ for 20 min, and the pellet washed twice with sterile PBS. The bacteria were re-suspended in ice-cold complete RPMI 1640 medium without antibiotics. The bacteria were then used for infecting cells according to the required MOI.

### Neutrophil purification

Human neutrophils were isolated from fresh venous blood obtained from healthy volunteers using a modified method adapted from Nauseef.^23^ All procedures were performed with the ethical approval of Glasgow University. The neutrophil purity was confirmed by rapid Romanowski staining before using for experiment and was > 90%.

### Neutrophil transfection

Neutrophils were washed and re-suspended in Gene pulser electroporation buffer (Cat. 165-2676; Bio-Rad Laboratories, Hercules, CA, USA) at approximately 5 × 10^6 ^cells/ml. ON-Target plus human *Atg 5* (9474) siRNA SMART pool (Cat. L-004374-00-0005; Thermoscientific RNAi Technologies, Paisley, UK) and control siRNA was added to a final concentration of 100 nM. The cell suspension was then transferred to 0.4-cm electrode gap sterile Gene-Pulser electroporation cuvettes (Cat. 165-2088; Bio-Rad) on ice. Electroporation of human neutrophils was performed using an X-cell Gene-Pulser machine (Bio-Rad) with settings of capacitance 1000 µF, resistance 1000 Ohm and voltage 250 v, with an exponential decay pulse. Cells were quickly washed with and re-suspended in complete RPMI 1640 medium after electroporation. The medium was supplemented with 10% heat-inactivated human serum and GM-CSF (10 ng/ml). The cells were then incubated at 37℃, 5% CO_2_ overnight. Viability was determined by 7-aminoactinomycin D staining and analysis by flow cytometry; viability remained at > 85% following electroporation.

### Rates of neutrophil phagocytosis and killing

Human neutrophils were isolated according the protocol described above. Cells were re-suspended at 1 × 10^6 ^cells/ml in complete RPMI 1640 medium containing heat-inactivated human serum. Cells were infected with *S. pneumoniae* at an MOI of 10 and incubated at 37℃ and 5% CO_2_. The same numbers of bacteria were grown in a second tube in the same medium as a control. The tubes were rotated end to end slowly and 1 ml infected neutrophils and bacterial suspension were collected at different time points (0–120 min). Infected neutrophils were centrifuged at 100 *g* for 5 min. The supernatant containing bacteria was collected in a tube and the neutrophil pellet was washed twice with PBS to remove any extracellular bacteria. The above procedure was repeated during each time point. Internalized bacteria were enumerated neutrophils by re-suspending the neutrophil pellet in 1 ml PBS containing 0.05% (w/v) saponin (Cat. 47036-50G-F; Sigma-Aldrich, St. Louis, MO, USA) on ice. The cell lysates were homogenized by gentle pipetting, serially diluted and incubated on the blood agar overnight. The number of bacteria in each sample from control, supernatant and cell lysates was enumerated by colony counting.

The method of Hampton et al. was used to derive the rates of phagocytosis and killing.^24^ Analysis was performed using a spread sheet available from the Hampton group at http://www.otago.ac.nz/free-radical/research/otago599670.html.

The uptake by phagocytosis and killing of bacteria by neutrophils occurs in series as described by equation (1):

(1) $$A\overset{k_{p}}{\to }B\overset{k_{k}}{\to }C$$

where *A* is the number of extracellular bacteria, *B* is the number of viable intracellular bacteria, *C* is the number of killed bacteria, *k_p_* is the rate constant of phagocytosis, and *k_ k_* the rate constant of killing. Assuming first-order kinetics gives the following differential equations:

(2) $$\frac{d[A]}{\mathrm{dt}}=k_{p}[A]$$

(3) $$\frac{d[B]}{\mathrm{dt}}=k_{p}[A]-k_{k}[B]$$

where *t* is time.

Solving:

(4) $$A=A_{0}e^{-k_{p}t}$$

where *A*_0_ is the original number of bacteria added.

Hence, *k_p_* can be derived from the slope of ln(*A*) vs. time.

(5) $$B=\frac{A_{0}k_{p}}{\left(k_{k}-k_{p}\right)}(e^{-k_{p}t}-e^{-k_{k}t})$$

*k_k_* can be determined at each time point by rearranging and solving equation [5] using the Lambert W function as described in the spread sheet referenced above. An average value is then calculated for all the different times.

LDH killing assay was performed using a Cytotox-96 kit (Promega, Southampton, UK).

### NET analysis

NETS were analysed using a modified protocol.^7^ Purified human neutrophils were seeded onto 13-mm sterile glass coverslips at a density of 4 × 10^5 ^cells per ml in RPMI containing 2% heat-inactivated human serum. Cells were infected with bacteria and left at 37℃ in 5% CO_2_ for 4 h before gentle fixation in 4% paraformaldehyde in PBS for 60 min. Cells were washed by inverting onto drops of PBS on parafilm, permeabilized with 0.5% Triton-X 100 using the same method and then blocked by inverting onto drops of 10% normal goat serum in PBS. After further washing they were then incubated with anti-neutrophil elastase (Abcam, Cambridge, UK) for 60 min followed by washing and secondary Ab visualization using the same method of inversion onto drops. DNA was counterstained using DAPI or Sytox Orange; bacteria were visualized by prior loading with eFluor 450 at 40 µM for 15 min (eBioscience, San Diego, CA, USA) before washing twice in PBS. Slides were viewed with a Zeiss Axiovert S100 microscope using OpenLab software (Perkin Elmer, Wokingham, UK). NET formation was quantified by direct visualization of the stained cells. Total cell counts were determined per field of view by their DNA stain. The cells that had undergone NETosis were classified as having extracellular material staining with neutrophil elastase. At least three views of 100 cells were counted and the mean percentage (± SEM) were calculated. We found that many of the neutrophils formed NETs from cells in close proximity, which would be underestimated by automatic counting procedures.

### Statistics

Differences between groups were tested using unpaired *t*-tests with Prism (GraphPad, La Jolla, CA, USA). A *P*-value < 0.05 was taken as significant.

## Results

### S. pneumoniae induces autophagy in human neutrophils

**Figure 1.**
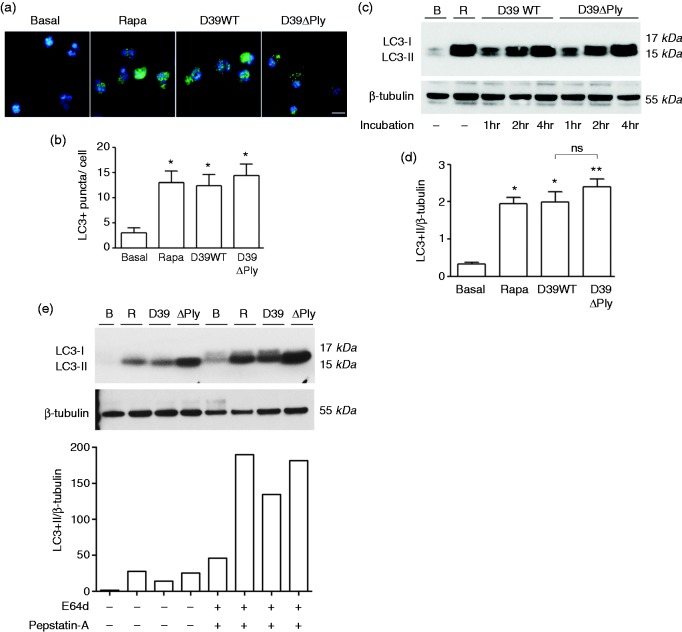
Neutrophils undergo autophagy following infection with pneumococcus. Neutrophils were left untreated (Basal, or ‘B’) or infected with wild type pneumococcal strain D39 (D39WT) or with the Ply-deficient mutant (D39ΔPly) at a multiplicity of infection of 10 for 4 h, or treated with Rapamycin (Rapa, or ‘R’) at a concentration of 50 µg/ml for 4 h. Accumulation of LC3 staining puncta was visualized by immunofluorescence as shown in (a), and quantified as shown in (b). Results are the means of triplicate determinations from at least 60 cells; error bars are SEM. (c) Lysates from cells treated as indicated for the times shown were immunoblotted for LC3 (upper panel) or β-tubulin (lower panel). Position of the different LC3 forms is shown to the left and molecular mass markers at the right. (d) Mean values of the ratio between density of LC3-II band to β-tubulin for treatments after 4 h, as indicated. Values are the means of 2–3 separate determinations; error bars are SEM. (e) as (c), but with addition of the inhibitors E64d (10 µg/ml) and pepstatin-A (10 µg/ml) as shown and incubation for 4 h. The lower panel shows values of the ratio between density of LC3-II band to β-tubulin for treatments as indicated. Experiment repeated with similar findings. In (b) and (d) significant differences from the basal level were determined by *t*-tests; *P < 0.05, **P < 0.01. Difference between D39WT and D39ΔPly was not significant (ns).

Firstly, we incubated purified human neutrophils with the D39 strain of pneumococcus for various times and assayed for the induction of autophagy by the detection of puncta containing the autophagocytic vacuole marker LC3. Following infection, there was a significant increase in the numbers of LC3 puncta per cell, both with wild type D39 and an isogenic mutant lacking pneumolysin (D39ΔPly), to the same degree as seen after treatment with rapamycin, an inhibitor of the mechanistic target of rapamycin (Figure 1a). Pneumolysin had no effect on autophagy induction. We also measured the conversion of LC3 from its native (LC3-I) to its lipidated form (LC3-II), a post-translational modification that occurs following induction of autophagy. Again, following infection with the D39 strain of pneumococcus we observed a marked increase in the levels of LC3-II relative to β-tubulin that increased over time, again with no significant difference between the wild type D39 and the D39ΔPly mutant (Figure 1b, c).

To ensure the changes in the levels of LC3-II were due to increased flux through the autophagocytic pathway rather than inhibition of autophagosome degradation, we repeated this assay in the presence of the lysosomal protease inhibitors. E64d and pepstatin A (Figure 1d). These inhibitors prevent lysosomal degradation and thus will reveal if the increase in LC3-II is due to increased formation of autophagosomes. In the presence of these inhibitors, we observed increased levels of LC3-II confirming an increased flux through the autophagocytic pathway following infection with D39. Importantly, over the time course (4 h) of these infections, cell viability remained > 90%, as measured by release of lactate dehydrogenase (data not shown). The increase in LC3-II levels in the presence of the lysosomal inhibitors was reproducible, although there was considerable variation in the absolute values of the LC3-II/β-tubulin ratio, precluding a valid statistical test of the increase observed. However, we also saw the same increase in LC3-II formation after D39 infection in the presence of bafilomycin, a different lysosomal inhibitor (data not shown).

### Autophagy following pneumococcal infection is inhibited by 3-methyladenine and requires *Atg5*

**Figure 2.**
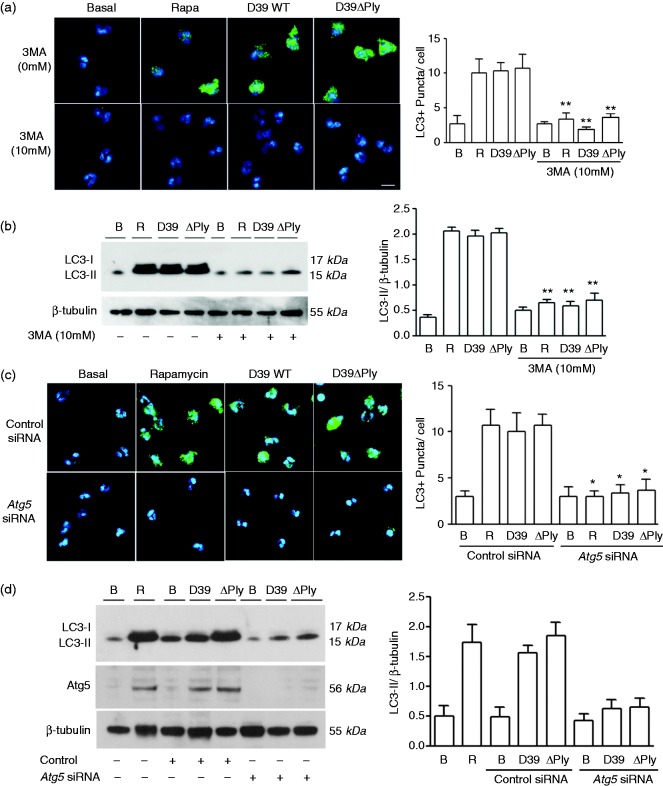
Autophagy following pneumococcal infection of neutrophils is dependent on type III PI3K and *Atg5*. Neutrophils were infected or treated with rapamycin as in Figure 1. (a) Immunofluorescence images of neutrophils treated as shown in the absence or presence of the type III PI3K inhibitor 3-methyladenine (3MA). Panel to the right shows quantification of the puncta as described in Figure 1. (b) Immunoblot of neutrophil lysates treated as shown and as described in Figure 1. Panel to the right shows quantification of repeats of the experiment as in Figure 1. (c) and (d) as (a) and (b), respectively, but with cells treated with siRNA to *Atg5* or control siRNA as indicated. Significant differences from the corresponding level in the absence of the inhibitor were determined by *t*-tests; *P < 0.05, **P < 0.01.

Next, we tested the effect of the type III phosphatidylinositol 3-kinase inhibitor (PI3K), 3-methyladenine (3MA)^25^ on autophagy following pneumococcal infection of neutrophils. The type III PI3K activates autophagy through production of phosphatidylinositol-3-phosphate that recruits essential autophagy proteins required for formation of the autophagocytic vacuole.^26^ Treatment of neutrophils with 3MA significantly abrogated the production of LC3 containing puncta in cells following pneumococcal infection (Figure 2a). It also significantly inhibited the conversion of LC3-I to LC3-II (Figure 2b), consistent with its known effects on inhibiting the classical pathway of autophagy.

To explore the mechanism of pneumococcal-induced autophagy further, we tested the effect of silencing the product of the essential autophagy gene *Atg5* on autophagy following pneumococcal infection. Compared with control siRNA, siRNA to *Atg5* significantly inhibited the formation of LC3 puncta following treatment of cells with rapamycin or following pneumococcal infection (Figure 2c). We also analysed the effect of *Atg5* silencing on the conversion of LC3-I to LC3-II. Following autophagy induction by rapamycin or pneumococcal infection, the Atg5 protein was significantly induced (Figure 1d, left panel), as has previously been described.^27^ Note these blots show the conjugate of Atg5 with Atg12 that forms on induction of autophagy.^28^ Again, compared with control siRNA, siRNA to *Atg5* produced a significant inhibition of Atg5 induction, and inhibited conversion of LC3-I to LC3-II (Figure 2d). In all these experiments, neutrophil viability remained > 90% (data not shown).

### Effects of pneumolysin and autophagy on the kinetics of phagocytosis and intracellular killing of pneumococci

**Figure 3.**
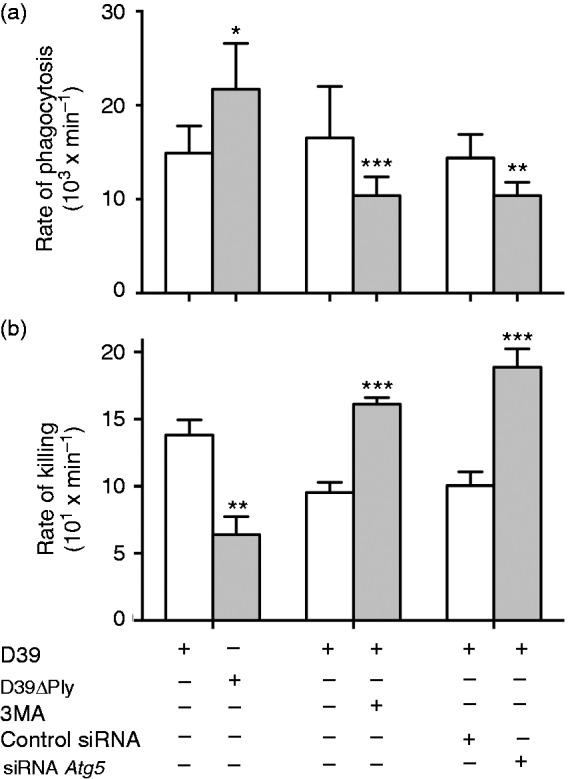
Rates of neutrophil phagocytosis and killing of pneumococci. Cells were treated as indicated and rates of phagocytosis (a) and killing (b) determined as described in ‘Material and methods’. Columns are the means from three (a) or five (b) determinations; error bars are 95% confidence limits (a) or SEM (b). Significant differences between the observations are shown (*t*-test): *P < 0.05; **P < 0.01; ***P < 0.001.

We then measured the effects of pneumolysin and autophagy on the rates of uptake and killing of the D39 pneumococcal strain by neutrophils. We used a single-step assay for measuring these parameters as developed by the Hampton group.^24,29,30^ This method uses a kinetic model to determine the rate constant of phagocytosis (*k_p_*) and killing (*k_k_*). Figure S1 shows the individual data values at the various time points in the experimental incubation of pneumococci with neutrophils used to derive the mean values for each constant. These mean values of *k_p_* and *k_k_* are shown in Figure 3. Firstly, we measured these kinetic constants in D39 WT and the D39ΔPly pneumolysin deficient mutant. *k_p_* was significantly increased in the D39ΔPly mutant, showing that pneumolysin normally acts to reduce the rate of phagocytosis by human neutrophils. *k_k_*, however, was significantly reduced in the D39ΔPly mutant, showing that the effect of pneumolysin is to increase the rate of intracellular killing.

Next, we calculated these rate constants for pneumococcal interaction with neutrophils in the presence of the autophagy inhibitor 3MA and with knockdown of the *Atg5* gene. The results from these determinations were very similar and showed that when autophagy was inhibited by either mechanism, the rate of phagocytosis was significantly reduced but the rate of intracellular killing was increased (Figure 3). These results are considered further in the discussion.

### NET formation following pneumococcal infection is dependent on autophagy but not on pneumolysin

**Figure 4.**
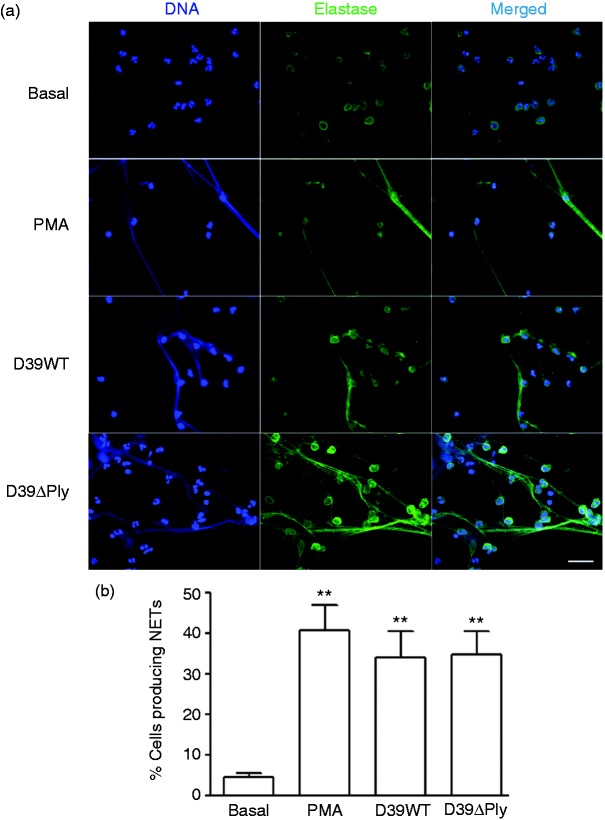
NET formation following pneumococcal infection of neutrophils. (a) Immunofluorescent images of neutrophils treated or infected as shown and stained for DNA (DAPI, blue), elastase (green) and merged. (b) quantification of NET formation as described in the ‘Materials and methods’. Results are means of triplicate determinations from views of at least 100 cells; error bars are SEM. Significant differences from basal levels are shown using the same symbols and statistical tests as Figure 1.

We analysed neutrophils following infection for the presence of NETS. Using immunofluorescent microscopy, we identified NETs by the presence of extracellular DNA coated with elastase, a neutrophil granule protein. Following incubation of neutrophils with D39, there was a marked and significant induction of NET formation, to a degree very similar to that produced by the known NET inducer, PMA (Figure 4a). We quantified the degree of NET formation both form the D39WT strain, as well as the D39ΔPly mutant (Figure 4b). Both strains showed a significant induction of NETs, but there no difference between them.

**Figure 5.**
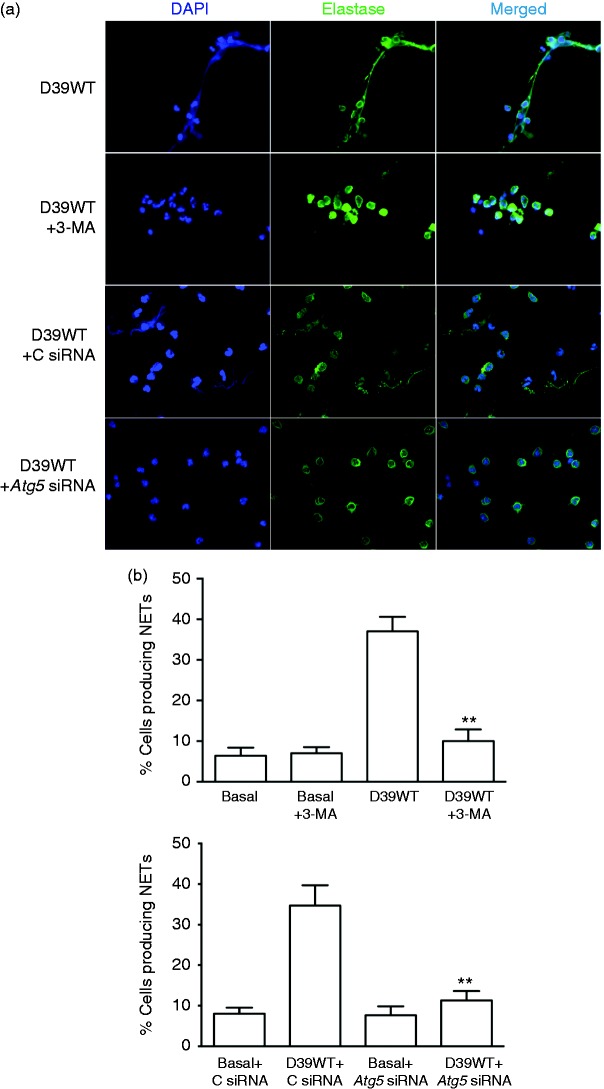
NET formation is dependent on autophagy. As in Figure 4, but cells treated with 3-MA or siRNA to *Atg5* or control siRNA (C, siRNA) as indicated. (a) Immunofluorescent staining. (b) Quantification of NET formation. Statistical analysis and symbols as in Figure 4.

We then determined the influence of autophagy on this NET formation, using the inhibitor 3MA as well as silencing of the *Atg5* gene (Figure 5). Using both methods (Figure 5b), we found that down-regulation of autophagy significantly reduced NET formation, almost to baseline values, demonstrating that autophagy is essential to NET formation following pneumococcal infection of human neutrophils.

### Influence of pneumolysin on pneumococcal trapping by NETs

**Figure 6.**
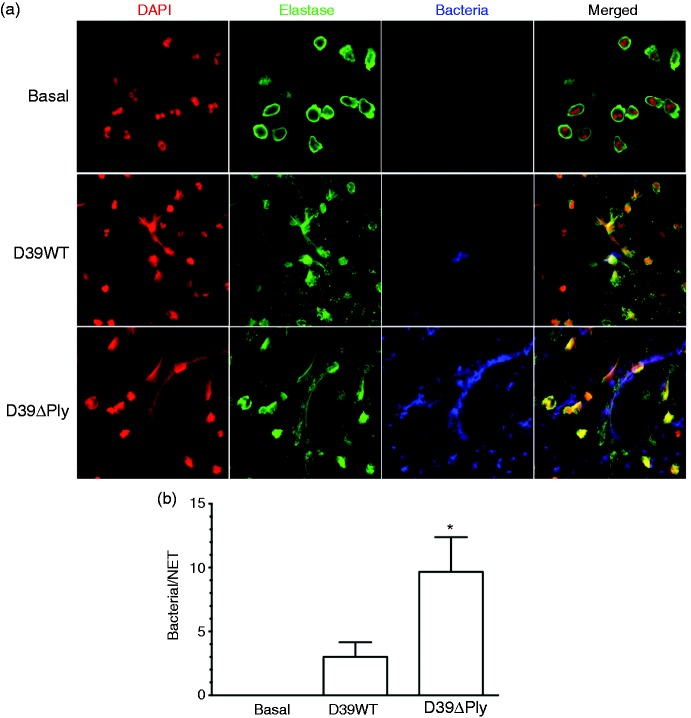
Pneumolysin inhibits bacterial capture by NETs. As in Figure 4, but with bacteria pre-loaded with eFluor 450 for visualization. (a) Immunofluorescent staining. (b) Quantification of the number of bacteria captured per NET. Columns are the means from three separate determinations of at least 20 NETs; error bars are SEM. Values for the Ply-deficient mutant (D39ΔPly) are significantly different from the D39WT (*t*-test, *P* < 0.05).

In the experiments analysing NET formation with the D39WT and D39ΔPly mutant, we noted there were apparently far fewer bacteria associated with NETs following infection with the D39WT strain compared with the D39ΔPly mutant. We quantified this difference (Figure 6), which confirmed there was a significant increase in the numbers of bacteria associated with NETs following infection of human neutrophils with the D39ΔPly mutant strain compared with the D39 WT. Thus, pneumolysin acts to inhibit pneumococcal association with NETs.

## Discussion

Neutrophils play a fundamental role in the innate immune response to pneumococcal infection. Our results highlight a number of important mechanisms underlying the interaction of the microbe with these cells. We show that pneumococci induce autophagy in human neutrophils in a classical type III PIK and Atg5-dependent fashion that is not dependent on pneumolysin. A previous report studying pneumococcal infection of A549 cells found that pneumolysin did play a role in autophagy induction.^17^ The transformed cell line A549 is said to synthesize large amounts of intracellular lipid,^31^ which may influence its interactions with the cholesterol-dependent cytolysin pneumolysin. This might account for the differences observed between A549 cells and our studies with primary human neutrophils.

Neutrophil phagocytosis of pneumococci is a key element of host defence. The data presented here show that pneumolysin inhibits the rate of phagocytosis by human neutrophils. A previous study showed that pneumolysin inhibited complement deposition on the surface of pneumococci and led to decreased phagocytosis.^32^ In our experiments, we used heat-inactivated human serum, thus suggesting effects on complement are not responsible. We also observed that pneumolysin increased the rate of intracellular killing within neutrophils. Although purified pneumolysin is reported to inhibit the neutrophil respiratory burst,^33,34^ release of pneumolysin from pneumococci by autolysis has been shown strongly to induce neutrophil oxygen radical production.^35^ This latter effect would account for the increased neutrophil killing in the presence of bacteria expressing pneumolysin observed here.

Our data show that autophagy enhances the rate of neutrophil phagocytosis of pneumococci. Previous reports have suggested that autophagy reduced the rate of macrophage phagocytosis of particles and mycobacteria.^36,37^ However, a novel form of phagocytosis, termed LC3-associated phagocytosis, has recently been described which utilizes the autophagocytic machinery to augment phagocytosis in a type III PI3K and Atg5-dependent fashion.^38^ This may account for the autophagy-enhanced phagocytosis observed here. The rates of intracellular killing, however, were reduced by autophagy in pneumococcal-infected neutrophils. This was surprising, as previous studies have suggested that autophagy augments neutrophil killing of the Gram-positive pathogen *Bacillus anthracis.*^39^ However, a number of pathogens have been shown to benefit from autophagy by nutrient acquisition;^40^ this may be the mechanism operative here.

We show that the pneumococcus is a potent stimulator of NET formation from neutrophils that is dependent on autophagy. Pneumolysin was not required for NET formation. A number of previous studies have suggested that autophagy is required for NET formation.^16–18^ The data reported here are the first demonstration that autophagy is essential for NET formation following pneumococcal infection of human neutrophils. The mechanism underlying the influence of autophagy on NET formation is not clear. In our study, NETosis was not associated with significant neutrophil death, as has been described by others.^9,10^ We speculate that release of nuclear and granule components from viable cells must utilize the autophagocytic pathway in some way.

We were surprised to find that pneumolysin inhibited NET capture of pneumococci. Previous studies have suggested that NETs capture but do not kill pneumococci.^11,41^ The mechanism by which pneumolysin acts to promote NET evasion by pneumococci is not clear. A recent report has found that pneumolysin is required for biofilm formation by the pneumococcus.^42^ We speculate that pneumolysin-induced biofilm formation may act to prevent capture of pneumococci by NETs and account for the observations reported here.

Clearance of pneumococci by neutrophils is a major component of the innate immune defence against pneumococcal colonization and infection. Our results show the complex interplay between pneumolysin expression, autophagy, phagocytosis, intracellular killing, and NET formation and capture of bacteria. Some of these responses have augmenting effects on one neutrophil process while down-regulating another. Pathogens such as the pneumococcus have evolved many sophisticated methods to evade immune clearance, and this has contributed to the complex interplay between the various factors highlighted in this study.
